# Self-reported impacts of the COVID-19 pandemic among people who use drugs: a rapid assessment study in Montreal, Canada

**DOI:** 10.1186/s12954-022-00620-w

**Published:** 2022-04-18

**Authors:** Nanor Minoyan, Stine Bordier Høj, Camille Zolopa, Dragos Vlad, Julie Bruneau, Sarah Larney

**Affiliations:** 1 Université de Montréal Hospital Research Centre (CRCHUM), 900 Rue Saint Denis, Montreal, QC H2X 0A9 Canada; 2 Department of Social and Preventive Medicine, École de Santé Publique, Université de Montréal, 7101 Ave Parc, Montreal, QC H3N 1X9 Canada; 3 Department of Family and Emergency Medicine, Faculty of Medicine, Université de Montréal, 2900 Boul. Édouard-Montpetit, Montreal, QC H3C 3J7 Canada

**Keywords:** COVID-19, People who use drugs, Harm reduction, Drug-related harms, Rapid assessment, Drug markets, Access to health services

## Abstract

**Background:**

People who use drugs (PWUD) are at high risk of experiencing indirect harms of measures implemented to curb the spread of COVID-19, given high reliance on services and social networks. This study aimed to document short-term changes in behaviours and health-related indicators among PWUD in Montreal, Canada following declaration of a provincial health emergency in Quebec.

**Methods:**

We administered a structured rapid assessment questionnaire to members of an existing cohort of PWUD and individuals reporting past-year illicit drug use recruited via community services. Telephone and in-person interviews were conducted in May–June and September–December 2020. Participants were asked to report on events and changes since the start of the health emergency (March 13, 2020). Descriptive analyses were performed.

**Results:**

A total of 227 participants were included (77% male, median age = 46, 81% Caucasian). 83% and 41% reported past six-month illicit drug use and injection drug use, respectively. 70% of unstably housed participants reported increased difficulty finding shelter since the start of the health emergency. 48% of opioid agonist treatment recipients had discussed strategies to avoid treatment disruptions with providers; 22% had missed at least one dose. Many participants perceived increased difficulty accessing non-addiction health care services. Adverse changes were also noted in indicators pertaining to income, drug markets, drug use frequency, and exposure to violence; however, many participants reported no changes in these areas. Among persons reporting past six-month injection drug use, 79% tried to access needle-syringe programmes during the health emergency; 93% of those obtained services. 45% tried to access supervised injection sites, of whom 71% gained entry.

**Conclusions:**

This snapshot suggests mixed impacts of the COVID-19 pandemic on PWUD in Montreal in the months following declaration of a provincial health emergency. There were signals of increased exposure to high-risk environments as well as deteriorations in access to health services. Pandemic-related measures may have lasting impacts among vulnerable subgroups; continued monitoring is warranted.

**Supplementary Information:**

The online version contains supplementary material is available at 10.1186/s12954-022-00620-w.

## Background

People who use illicit drugs (PWUD) are particularly vulnerable to experiencing direct and indirect harms of the COVID-19 pandemic [1]. Substance use disorder and chronic physical conditions including smoking and respiratory issues are common in this population, increasing risk of complications and mortality in the event of SARS-CoV-2 infection [2, 3]. PWUD often depend on low-threshold health and social services to obtain medical care, harm reduction supplies, and food, income, or shelter [4]. Some rely on social interactions to meet these daily needs, as well as to obtain drugs and consume them safely [5]. Service disruptions and reduced opportunities for social contact in the context of social distancing measures may thus exacerbate daily crises in this group during respiratory infection pandemics [4, 6].

To mitigate some of the anticipated consequences of the COVID-19 pandemic on PWUD, Canadian health authorities facilitated adaptations to existing services (e.g. by increasing allowances for take-home dosing of opioid agonist treatment) and implemented emergency measures (e.g. makeshift shelters) following health emergency declarations in the provinces and territories [7–9]. Nevertheless, increases in drug-related harms were subsequently noted in several settings [10–12]. Sharply increasing overdose rates in mid-2020 provided stark evidence of worsening conditions for PWUD [11, 13], with closures of harm reduction sites, drug market-related changes due to travel restrictions, as well as lockdown mandates driving people to use drugs alone proposed as contributing causes [14]. In Montreal, Quebec, harm reduction sites were either closed or operating with reduced capacity and/or hours in the weeks and months following the provincial health emergency declaration on March 13, 2020 [15, 16], raising concerns about possible outbreaks of hepatitis C virus/HIV infection in addition to elevated overdose risk [17].

Prior research suggests that events causing widespread disruption (“Big Events”) [18] carry acute consequences for PWUD [19–22]. In mid-2020, our team conducted a rapid review of this literature, identifying several anticipated changes, including the reorganization of drug markets, shifts in drug use patterns, as well as disruptions to health service provision, based on studies conducted in the wake of major hurricanes, the 2008 global financial crisis, and heroin shortages in different settings [23]. Findings were synthesized into a conceptual model describing possible consequences of the COVID-19 pandemic across a range of determinants known to influence health and risk behaviours among PWUD [23]. Importantly, the review identified no studies assessing the impacts of previous respiratory infection pandemics on PWUD.

During that same period, we initiated a rapid assessment study to document short-term impacts of the pandemic on the health and well-being of PWUD in Montreal. Specific aims were to characterize (1) COVID-19 knowledge, concerns, and potential exposure; (2) changes in ability to meet health- and survival-related needs; (3) changes to drug use patterns and risk environments including drug markets; (4) adverse health events and drug-related risk behaviours during the pandemic. We report findings on the latter three aims relating to indirect impacts.

## Methods

The rapid assessment study was initiated in May 2020. We contacted current members of a long-standing cohort study of people who inject drugs (PWID) in Montreal (HEPCO) [24] and additionally recruited individuals reporting past-year illicit drug use from a variety of community service sites. Individuals eligible for inclusion in HEPCO report past six-month injection drug use, are aged 18 years or older, and live in the greater Montreal region. We contacted participants who had a signed consent form on file for the most recent iteration of the cohort (beginning in 2017) and invited them to complete a one-time questionnaire focusing on their experiences during the COVID-19 pandemic. Community services sites were situated on the Montreal Island and were known to provide services to people who use drugs. Recruitment and interviews were facilitated by a community liaison with lived experience.

A structured questionnaire was developed with reference to a systematic review of health needs assessments in disaster contexts [25]. Unless otherwise specified, question wording assessed changes since declaration of the health emergency in Quebec. The comparator period was usually undefined; most questions asked participants to compare their behaviour/situation during the health emergency to their usual state.

Institutional review board approval was obtained at the Centre Hospitalier de l’Université de Montréal (CHUM). Study staff were experienced in interviewing members of this population, as they were implicated in other projects within the research team. The rapid design of the study precluded extensive piloting and revision of the questionnaire; however, a short training period allowed interviewers to practise questionnaire administration and understand how to code responses. Interviews were conducted in compliance with sanitary measures in effect at the time. Given lockdown restrictions, data collection in May–June 2020 was limited to telephone interviews with HEPCO participants; verbal informed consent was required. Interviews conducted from September to December 2020 could take place remotely or on-site at the CHUM or within community services. Participants in the latter setting had the option to borrow a study phone to communicate with interviewers. Participants received 20$ CAD as compensation for their time.

### Analyses

Descriptive statistics were computed, restricting to relevant subgroups where indicated to aid interpretation (e.g. injection-related questions among PWID, housing access questions among the unstably housed subset).

## Results

Of 405 HEPCO participants with available contact information, 129 completed the rapid assessment questionnaire (101 in May–June 2020, 28 in September-December 2020). An additional 99 participants were recruited via community services. The current analytic sample excludes one participant who terminated the interview soon after starting, and duplicate data from a participant mistakenly interviewed twice. Table 1 presents characteristics of the 227 included participants. Three-quarters of the sample self-identified as male, 81% were Caucasian, and over one-third were unstably housed. 41% reported past six-month injection drug use. Opioid and cocaine injection, as well as polydrug use, were common among this subset.

**Table 1:** Participant characteristics (*N* = 227)

	*n* (%)
Age, median [*Q*1–*Q*3]	46 [39–56]
*Gender*	
Male	174 (76.7)
Female	49 (21.6)
Other	4 (1.8)
*Ethnicity*^a^	
White/Caucasian	182 (80.9)
Indigenous	12 (5.3)
Other	31 (13.8)
Recent unstable housing^b^	83 (36.6)
Survival^c^ income source(s) in the 3 months prior to pandemic	40 (17.6)
Diagnosed with ≥ 1 chronic physical condition^d^	133 (58.8)
Living with a mental disorder, or a serious mental health problem	94 (41.4)
Opioid agonist treatment, current	69 (30.4)
Alcohol use, past 6 months	159 (70.0)
Illicit drug use, past 6 months	188 (82.8)
*Most frequently used drug, past 6 months*	
Opioid	35 (15.4)
Stimulant	93 (41.0)
Alcohol	36 (15.9)
Cannabis	52 (22.9)
Ketamine	2 (0.9)
None	7 (3.1)
Injection drug use, past 6 months	94 (41.4)
*Drugs injected*^e^	
Heroin	52 (55.3)
Prescription opioids	32 (34.0)
Cocaine	47 (50.0)
Amphetamine	13 (13.8)
Both opioid & stimulant	27 (28.7)
Other	5 (5.3)

^a^2 missing values

^b^Question pertained to the 6 months preceding the interview in May–June 2020, and the period since the pandemic (ranging from 6 to 9 months prior to the interview) in September–December 2020

^c^Reporting income from informal or street-based income generation activities, e.g. panhandling, sex work, recycling, pawning

^d^Self-reported lifetime diagnosis of: lung disease, diabetes, cardiovascular disease, chronic liver or kidney disease, HIV, HCV, or other condition affecting immune system

^e^% calculated among people reporting past six-month injection drug use (*n* = 94)

### Changes in ability to meet survival-related needs (Table 2)

**Table 2:** Changes in ability to meet survival-related needs (*N* = 227)

	*n* (%)
Any change in living situation	73 (32.2)
*New living situation is:* ^a^	
Worse	26 (35.6)
Better	34 (46.6)
About the same	11 (15.1)
Don’t know/missing	2 (2.7)
Became homeless as a result of the COVID-19 crisis	42 (18.5)
*Change in ease of finding a place to stay, among unstably housed (N* = *83)*	
More difficult	58 (69.9)
Easier	2 (2.4)
Same as usual	11 (13.3)
Don’t know/not applicable	12 (14.5)
*Amount of monthly income able to generate or access has*…^b^	
Decreased	82 (36.1)
Increased	38 (16.7)
Not changed	105 (46.3)
Don’t know	2 (0.9)

^a ^% calculated among those experiencing any change in living situation (n = 73)

^b^1 missing value

One-third of the sample had experienced a change in living situation since the start of the health emergency. While 36% qualified this as a change for the worse, almost half reported an improvement. Almost one-fifth of the overall sample said they had become homeless because of the pandemic. A majority of participants reporting recent unstable housing (see Table 1 for definition) found that it had become more difficult to find a place to stay.

Almost two-thirds of the sample reported an increase or no change in the amount of monthly income they were able to generate or access, while roughly one-third reported a decrease. Additional file 1: Table S1 presents reported income sources during the health emergency versus the three months preceding the declaration. Most participants had already been receiving welfare and/or governmental support, with little change since the declaration. In contrast, the proportion reporting full-time work fell from 19 to 9%. Overall, 18% and 14% of participants reported ≥ 1 survival income generation activity in the pre- and post-pandemic period, respectively. 15% reported income from pandemic relief measures.

### Access of non-addiction health care services and opioid agonist treatment (Table 3)

**Table 3:** Access to health services and opioid agonist treatment

Overall sample	*N* = 227
	*n* (%)
*Perceived access to health clinics*	
Harder	119 (52.4)
Easier	10 (4.4)
Same as usual	42 (18.5)
Don’t know	55 (24.2)
*Perceived access to emergency departments*	
Harder	95 (41.9)
Easier	6 (2.6)
Same as usual	44 (19.4)
Don’t know	81 (35.7)
*Perceived access to other hospital services*	
Harder	94 (41.4)
Easier	3 (1.3)
Same as usual	35 (15.4)
Don’t know	93 (41.0)
*Perceived access to mental health services*	
Harder	82 (36.1)
Easier	2 (0.9)
Same as usual	34 (15.0)
Don’t know	106 (46.7)

OAT recipients	*N* = 69
	*n* (%)
Discussed ≥ 1 strategy to favour OAT continuity with prescriber	33 (47.8)
*Strategies discussed*^a^:	
Initiation of take-home/carry doses	15 (45.5)
Allowing longer take-home and prescription intervals	17 (51.5)
Arrangements to have medication delivered	9 (27.2)
Initiation of suboxone, methadone or other forms of treatment for opioid use disorder	7 (21.2)
Dosage modification	6 (18.2)
Initiation of co-medication (e.g. clonidine, loperamide)	2 (6.1)
Missed ≥ 1 OAT dose due to service disruptions	15 (21.7)

OAT, opioid agonist treatment

^a^% calculated among people reporting at least one strategy (n = 33)

Participants commonly perceived that non-addiction health care services (clinics, emergency departments, other hospital services, mental health services) had become harder to access. Of 69 participants receiving opioid agonist treatment (OAT) at the time of their study interview, approximately half had discussed strategies to maintain treatment continuity with their provider(s) (see Table 3). One-fifth had missed ≥ 1 dose due to COVID-19-related service disruptions, and one participant reported ceasing OAT for reasons related to the health emergency. Participants were generally satisfied with adaptations made by OAT providers (Additional file 1: Figure S1).

### Access of drug or alcohol treatment and harm reduction services (Additional file 1: Table S2)

Few participants (*n* = 34, 15%) tried to access drug/alcohol treatment during the health emergency, and just half of these were able to receive services. Among people reporting past six-month injection drug use (*N* = 94), 79% tried to access needle-syringe programmes, the majority of whom (93%) were able to obtain services/materials. 45% tried to access SIS, of whom 71% were able to gain entry. 18% and 23% tried to access naloxone and drug checking, respectively, of whom over 90% were able to obtain services.

### Changes in risk environments, drug markets and drug use patterns (Table 4)

**Table 4:** Changes in risk environments, drug markets and drug use patterns

Overall sample	*N* = 227
	*n* (%)
*Frequency of witnessing physical violence has*…	
Increased	93 (41.0)
Decreased	19 (8.4)
Not changed	94 (41.4)
Don't know/refused/not applicable^a^	21 (9.3)
*Frequency of experiencing physical violence has*…	
Increased	66 (29.1)
Decreased	14 (6.2)
Not changed	122 (53.7)
Don't know/refused/not applicable^a^	25 (11.0)
*Frequency of being “jacked up”/harassed by the police has*…	
Increased	48 (21.2)
Decreased	14 (6.2)
Not changed	14 (6.2)
Don't know/refused/not applicable^a^	27 (11.9)
*Thinks the amount of fentanyl in the market has*…	
Increased	86 (37.9)
Decreased	5 (2.2)
Not changed	35 (15.4)
Doesn’t know	101 (44.5)
Has heard of new drugs coming into the market	42 (18.5)
*Overall use of non-injection drugs has*…	
Increased	81 (35.7)
Decreased	52 (22.9)
Not changed	94 (41.4)
*Overall use of injection drugs has*…^b^	
Increased	36 (15.9)
Decreased	32 (14.1)
Not changed	158 (69.6)

Subset reporting an illicit drug as their most frequently used substance during the last 6 months	*N* = 132
	*n* (%)
*Availability of most frequently used drug has*…	
Increased	12 (9.1)
Decreased	43 (32.6)
Not changed	73 (55.3)
Don’t know/refused	4 (3.0)
*Price of most frequently used drug has*…	
Increased	56 (42.4)
Decreased	3 (2.3)
Not changed	71 (53.8)
Don’t know/refused	2 (1.5)
*Quality of most frequently used drug has*…	
Increased	3 (2.3)
Decreased	55 (41.7)
Not changed	68 (51.5)
Don’t know/refused	6 (4.5)
*Difficulty of obtaining credit from dealers has*…	
Increased	33 (25.0)
Decreased	10 (7.6)
Not changed	53 (40.2)
Don’t know/refused	36 (27.3)
Any change in the drug market indicators above	109 (82.6)
*Changes in the drug market have impacted what is consumed (or how it is consumed)*	43 (39.4)
Impacts cited:^c^	
Cut down consumption in general	20 (46.5)
Switched substances	12 (27.9)
Increased consumption in general	5 (11.6)
Drank more alcohol	2 (4.7)
Used more cannabis	2 (4.7)
Switched to injection	1 (2.3)
Other	10 (23.3)

^a^Not applicable (N/A) response option added in September–December

^b^1 missing value

^c^% calculated among those experiencing any drug market related change (*n* = 43)

Many participants reported increased frequencies of witnessing (41%) or experiencing (29%) physical violence and experiencing police harassment (21%) since the start of the health emergency, with smaller numbers reporting decreases across these indicators (6–8%). 38% of the sample perceived an increased presence of fentanyl in the drug supply, while 19% had heard of substances newly available on the market. 42% and 70% reported no change in their overall use of non-injection and injection drugs, respectively. Increases and decreases in injection drug use were reported with similar frequency, whereas slightly more participants increased versus decreased their non-injection drug use.

Among participants reporting an illicit drug as their most frequently used substance during the last six months, approximately half reported no change in the availability, price, or quality of that drug, while 33–42% perceived negative impacts in these areas. One-quarter reported increased difficulty obtaining credit from dealers. Of those perceiving any such change(s) in the drug market, over one-third reported that these had influenced which drugs they consumed and/or how they consumed them (further details in table).

### Drug-related risk behaviours and adverse health outcomes (Table 5)

**Table 5:** Drug-related risk behaviours and adverse health outcomes

Overall sample	*N* = 227
	*n* (%)
Bought drugs from a new dealer^a^	106 (46.7)
*Frequency of withdrawal/dopesickness has*…^a^	
Increased	20 (11.9)
Decreased	6 (3.6)
Not changed	142 (84.5)
Overdosed by accident	19 (8.4)
*Frequency of overdose has*…	
Increased	9 (4.0)
Decreased	9 (4.0)
Not changed	207 (91.2)
Don’t know	2 (0.88)
Has been feeling more depressed or anxious than usual	133 (58.6)

Subset reporting past six-month injection drug use	*N* = 94
	*n* (%)
*Frequency of injecting drugs alone has*…	
Increased	12 (12.8)
Decreased	15 (16.0)
Not changed	67 (71.3)
*Frequency of injecting drugs in public has*…^b^	
Increased	14 (15.2)
Decreased	13 (14.1)
Not changed	65 (70.7)
Injected with people outside of usual partners^b^	11 (11.8)
Injected with used needle-syringe or other injection materials^b^	13 (14.3)

^a^Denominators exclude participants reporting “don’t know” or “not applicable” (*n* = 68); many participants felt this question did not pertain to them, as they did not use opioids

^b^Denominators exclude missing values (maximum *n* = 3)

Almost half the sample reported having bought drugs from a new dealer since the start of the health emergency. Frequency of injecting drugs alone, injecting drugs in public, and experiencing withdrawal remained largely unchanged, while 59% reported increases in feelings of depression/anxiety.

## Discussion

This rapid assessment study documented short-term impacts of the COVID-19 pandemic on PWUD in Montreal, Canada, focusing on perceptions and experiences following the declaration of a public health emergency in Quebec in March 2020. The study, which collected data up to December 2020, has relevance for pandemic response planning given the paucity of literature examining the impacts of such crises on PWUD through primary data collection [23].

Previous studies assessing outcomes of other events causing widespread disruption (e.g. hurricanes, economic crises, heroin shortages) have identified important adverse impacts on drug markets, service provision, drug use patterns, and drug-related risk behaviours and harms [23]. Impacts documented in the current study were mixed, but suggested limited access to services and deteriorating risk environments for vulnerable subgroups, corroborating findings from qualitative research in Canada [26].

### Situating findings within the big events evidence base

We discuss specific findings with reference to our previously developed conceptual model outlining hypothesized consequences of the COVID-19 pandemic on PWUD, based on evidence from other “Big Events” (Additional file 1: Fig. S2) [23]. We observed several adverse outcomes that were consistent with our model. Indicators pointed to disruptions in the drug market and increased violence and policing following declaration of the health emergency. Many participants perceived reductions in drug availability and quality, as well as increased prices, with these factors influencing consumption patterns in some cases. A noteworthy portion of the sample experienced a loss of income, with both full-time employment and street-based income opportunities appearing to be impacted. Over half reported increases in anxiety/depression symptoms.

The study questionnaire only assessed harm reduction service access during the health emergency (rather than assessing *changes* in access). Access to supervised injection sites, naloxone, and drug checking appeared sub-optimal. In contrast, participants on OAT were generally able to access their treatment, and needle-syringe programme access appeared high. Anecdotal evidence suggests, however, that the provision side was heavily affected, particularly in the immediate aftermath of the declaration, with certain harm reduction sites temporarily closing or severely limiting operations. Furthermore, although access among OAT enrollees appeared adequate, we did not estimate changes in the rate of OAT initiation in individuals with opioid use disorder, an important outcome for future longitudinal research. Service side administrative data may shed further light on these trends.

Contrary to our expectations, frequency of withdrawal and non-fatal overdose remained largely unchanged, and impacts on additional risk behaviours (i.e. injecting drugs alone / in public) appeared minimal when participants were asked to compare the time since the start of the health emergency to their usual situation.

### Omitted dimensions

The Big Events conceptual model developed for COVID-19 did not outline possible impacts on non-addiction health care services (e.g. emergency departments), which are nevertheless frequently utilized by PWUD [27], and were perceived as having become harder to access by our participants. The model did not propose impacts on housing, which is both a crucial health determinant among PWUD [28], and a central issue for pandemic control [29]. Our indicators suggested that most unstably housed individuals experienced greater difficulty finding shelter during the assessed period, and one-fifth of the sample reported having become homeless as a result of the health emergency. The omission of housing and non-addiction health care service impacts from our conceptual model, which was also highlighted in a recent study [30], was due to the lack of studies focusing on respiratory infection pandemics in the Big Events evidence base [23]. Nevertheless, these dimensions remain absent from a recent framework synthesizing how the unintended impacts of COVID-19 (with respect to overdose risk) were discussed in commentaries, reviews, and original studies published in scientific journals up to December 2020 [31]. There is thus a need to more explicitly integrate these upstream determinants in future research and when elaborating pandemic response plans.

### Public health implications

A key takeaway from the study is that the COVID-19 pandemic has had variable impacts on PWUD in Montreal. Though we documented adverse effects on housing, income, drug markets and perceived access to health services, many respondents also reported experiencing little to no change in these areas. It is important to note that this perceived lack of change does not necessarily signify a lack of need for support during this and other Big Events; rather, it may signify that PWUD are often navigating difficult circumstances, including precarious housing and income, as well as environments where their physical presence is not welcome. The COVID-19 pandemic may not have been as strongly disruptive among PWUD as it has been for the general population. Instead, as suggested in recent qualitative work [32], it may be one of several ongoing crises that PWUD contend with. Removing existing barriers to health and social services remains an important endeavour to reduce inequities experienced by members of this group.

Given the negative outcomes documented among certain subgroups, it is crucial that the diversity of drug-using populations be considered during disaster responses, and that adaptations to services be rapid and guided by detailed response plans. Approaches should be tailored to reach the most vulnerable, who may not have the social or financial capital (including the technological means [33]) to access support/services essential for their survival. A wide range of response plans may be needed depending on the nature of disruptions. Plans should include provisions that services requiring physical presence (e.g. supervised injection sites) remain open at all times, thus preventing harms that outright closures may engender.

A critical area of vulnerability that requires greater consideration in such plans is housing instability, which is common among PWUD. In the case of respiratory infection pandemics such as COVID-19, reduced capacity in shelters due to crowding concerns, and reduced opportunities for informal arrangements due to social distancing measures, may lead to increased exposure to street-based environments consistently associated with high-risk behaviours and harms. Housing action plans which consider substance use, and novel Housing First approaches which co-situate mental and physical health supports, are urgently needed, as they directly modify the underlying risk environments known to drive harms among PWUD [34]. Initiatives implemented in response to COVID-19 (e.g. [35]) should be replicated, with designs allowing assessment of the active ingredients driving observed effects [36].

Socioeconomic instability due to decreased income generation opportunities, and shifting drug markets due to changes in global drug trafficking [14], may also exacerbate vulnerability among PWUD, both indirectly, by affecting drug use patterns, and directly, through increased risk of overdose from contaminated or increasingly potent supplies [37–39]. Preventing harms, both in the immediate wake of Big Events and in the longer term, requires continued operation and funding of harm reduction services and monitoring of the drug supply, including through scale-up of drug checking services [40], which were accessed by a limited number in our sample. The pandemic has been a window of opportunity to implement and evaluate innovative responses such as providing a safe supply of pharmaceutical drugs [40, 41], and adopting flexible OAT dispensation regimens, both of which reduce exposure to the illicit supply (and the costs and contaminants associated with it) [42]. It is important that the knowledge generated during this time be translated into progressive policies for the longer term.

Finally, health care service disruptions and reorganizations, including longer-term shifts towards telehealth, may disproportionally threaten the overall health of PWUD by setting back efforts to screen for, treat and eliminate hepatitis C virus in this population [43, 44], in addition to diagnosing and treating prevalent comorbidities such as mental health disorders, HIV, and other chronic diseases. As was true prior to the pandemic, services should be available in low-threshold settings to counter the marginalization of PWUD, and barriers such as stigmatizing language should be minimized within mainstream services to prevent disengagement from tertiary care [45].

### Limitations

PWUD constitute a “hidden” group; no sampling frame is available to recruit members of this population. Our convenience sample may therefore not represent all PWUD in Montreal, nor PWUD from other urban settings with different drug use and sociodemographic profiles. Samples sizes for some subgroups of PWUD (e.g. OAT recipients) were small. Findings may reflect subjective perceptions or secular trends in events. The study was conducted against an ever-evolving backdrop of public health restrictions. As such, findings constitute a snapshot of experiences in the months following the declaration of a health emergency in Quebec and may underestimate acute impacts felt in its immediate aftermath. Furthermore, the impacts of curfew measures implemented after our study (from January to May 2021) are unclear. Service access during peak (post-curfew) hours and the policing of service seekers were major concerns during that period [17, 46].

## Conclusions

The current snapshot suggests mixed impacts of the COVID-19 pandemic on PWUD in Montreal, with signals of increased exposure to high-risk environments and deteriorations in access to health services and housing. Changes were not experienced uniformly by all PWUD. Continuity of low-threshold service provision and in-person interactions may be critical to ensure survival among the most vulnerable. Measures to curb COVID-19 spread may have lasting drug-related harms even once they are lifted; continued monitoring, and greater consideration of the structural drivers of these harms, is warranted.

## Supplementary Information

**Additional file 1**. PDF document containing the following supplementary figures & tables: **Figure S1.** Satisfaction with adaptations made by pharmacy/medical team to deliver OAT during the health emergency; **Figure S2.** Anticipated outcomes of the COVID-19 pandemic based on a rapid review of select "Big Events", as reported in Zolopa et al 2021; **Table S1.** Changes in income sources; Table S2. Access to drug/alcohol treatment and harm reduction during the health emergency.

## Data Availability

The data that support the findings are available on request due to privacy/ethical restrictions. Data are not publicly available as individual-level information may identify participants.

## Abbreviations

HIV: Human immunodeficiency virus
OAT: Opioid agonist treatment
PWID: People who inject drugs
PWUD: People who use drugs

## Code availability

Code for statistical analysis available upon request.
