# Supplementary material for: Self-reported impacts of the COVID-19 pandemic among people who use drugs: a rapid assessment study in Montreal, Canada
Source: Harm Reduct J. 2022 Apr 18;19:38. doi: 10.1186/s12954-022-00620-w (PMC9013973; doi:10.1186/s12954-022-00620-w)
Supplement: Supplementary file 1 — Additional file 1. PDF document containing the following supplementary figures & tables: Figure S1. Satisfaction with adaptations made by pharmacy/medical team to deliver OAT during the health emergency; Figure S2. Anticipated outcomes of the COVID-19 pandemic based on a rapid review of select "Big Events", as reported in Zolopa et al 2021; Table S1. Changes in income sources; Table S2. Access to drug/alcohol treatment and harm reduction during the health emergency. [file 12954_2022_620_MOESM1_ESM.pdf]

Supplementary materials for the manuscript entitled "Self-reported impacts of the COVID-19 pandemic among people who use drugs: a rapid assessment study in Montreal, Canada" published in the Harm Reduction Journal. Authors: Nanor Minoyan, Stine Bordier Høj, Camille Zolopa, Dragos Vlad, Dragos Vlad, Julie Bruneau, Sarah Larney.

Figure S1. Satisfaction with adaptations made by pharmacy/medical team to deliver OAT during the health emergency

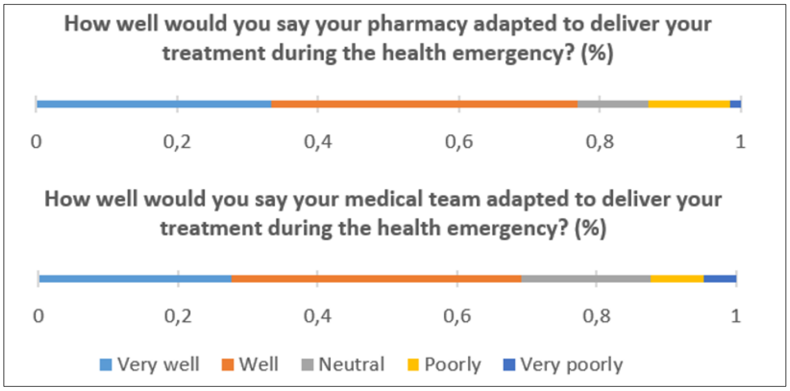

Figure S2. Anticipated outcomes of the COVID-19 pandemic based on a rapid review of select "Big Events," as reported in Zolopa et al 2021

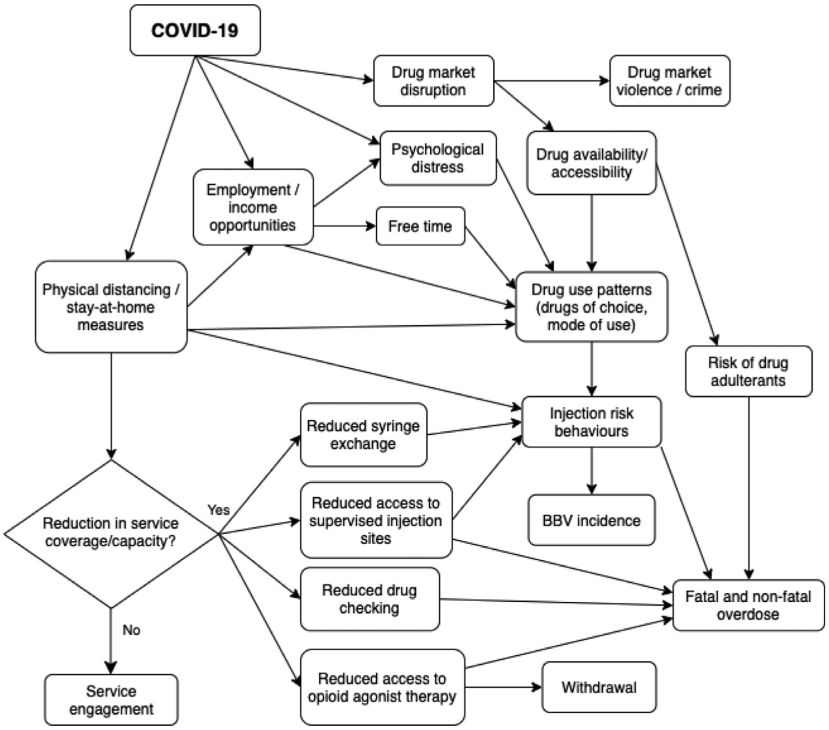

Fig. 5. Proposed risk pathway map for the COVID-19 pandemic.

Table S1. Changes in income sources (N=227)

	Using this income source:	
**Income source:**<br>**(n reporting source in both periods)**	**In the 3 months prior to the health emergency declaration - n (%)**	**Since the health emergency declaration n (%)**
COVID-19 government relief	-	33 (14.5)
Welfare (n=137)	145 (63.9)	151 (66.5)
Government loans/bursaries or other (para)governmental income (n=29)	29 (12.8)	36 (15.9)
Employment insurance (n=5)	7 (3.1)	10 (4.4)
Occasional or part-time work (n=17)	44 (19.4)	32 (14.1)
Full-time work (n=17)	44 (19.4)	21 (9.4)
Support from friends or family (n=9)	11 (4.8)	10 (4.4)
Any “survival” source	40 (17.6)	31 (13.7)
Panhandling (n=12)	16 (7.0)	17 (4.5)
Sex work (n=3)	5 (2.2)	3 (1.3)
Theft/fraud (n=0)	8 (3.5)	0 (4.0)
Selling personal items (n=1)	3 (1.3)	1 (0.4)
Selling drugs (n=5)	8 (3.5)	9 (4.0)
Recycling (n=2)	5 (2.2)	2 (0.9)
“Squeegee” (n=0)	2 (0.9)	0 (0)
No income source (n=0)	4 (1.8)	3 (1.3)

Table S2. Access to drug/alcohol treatment and harm reduction during the health emergency

**OVERALL SAMPLE (N=227)**		
	n	%
**Access to drug/alcohol treatment**		
Did not try to access	190	85%
Tried but was unable to access	17	8%
Tried and was able to access	17	8%
% failed access attempts		*50%*
**SUBSET REPORTING PAST-SIX MONTH INJECTION DRUG USE (N=94)**		
**Access to needle-syringe programs**		
Did not try to access	20	21%
Tried but was unable to access	5	5%
Tried and was able to access	69	73%
% failed access attempts:		*7%*
**Access to supervised injection sites**		
Did not try to access	52	55%
Tried but was unable to access	12	13%
Tried and was able to access	30	32%
% failed access attempts:		*29%*
**Access to naloxone**		
Did not try to access	72	77%
Tried but was unable to access	1	1%
Tried and was able to access	21	22%
% failed access attempts:		*5%*
**Access to drug checking**		
Did not try to access	77	82%
Tried but was unable to access	1	1%
Tried and was able to access	14	15%
% failed access attempts:		*7%*
